# *Moringa oleifera*: A Review on the Antiproliferative Potential in Breast Cancer Cells

**DOI:** 10.3390/cimb45080434

**Published:** 2023-08-18

**Authors:** Malebogo M. Moremane, Beynon Abrahams, Charlette Tiloke

**Affiliations:** Department of Basic Medical Sciences, School of Biomedical Sciences, Faculty of Health Sciences, University of the Free State, Bloemfontein 9301, South Africa; 2018305264@ufs4life.ac.za (M.M.M.); abrahamsbr@ufs.ac.za (B.A.)

**Keywords:** apoptosis, breast cancer, oxidative stress, antiproliferative, *Moringa oleifera*

## Abstract

The global burden of female breast cancer and associated deaths has become a major concern. Many chemotherapeutic agents, such as doxorubicin, have been shown to have adverse side effects. The development of multi-drug resistance is a common occurrence, contributing to chemotherapeutic failure. The resistance of breast cancer cells to drug treatment leads to a decline in the treatment efficacy and an increase in cancer recurrence. Therefore, action is required to produce alternative drug therapies, such as herbal drugs. Herbal drugs have been proven to be beneficial in treating illnesses, including cancer. This review aims to highlight the antiproliferative potential of *Moringa oleifera* (MO), a medicinal tree native to India and indigenous to Africa, in breast cancer cells. Although MO is not yet considered a commercial chemopreventive drug, previous studies have indicated that it could become a chemotherapeutic agent. The possible antiproliferative potential of MO aqueous leaf extract has been previously proven through its antioxidant potential as well as its ability to induce apoptosis. This review will provide an increased understanding of the effect that MO aqueous leaf extract could potentially have against breast cancer.

## 1. Introduction

Non-communicable diseases signify almost 71% of worldwide mortalities, with cancer and cardiovascular diseases accounting for 9.3 and 17.9 million deaths, respectively [1]. Cancer is a condition in which some of the body’s cells proliferate uncontrollably, and it has been observed to have the ability to metastasize in various body parts [1]. Additionally, it can regenerate in different sides of the human body and is made up of trillions of cells. Human cells have a lifespan, and they are replaced through cell division to form new cells as the body needs them [2]. When there is aging or injury of the cells, apoptosis takes place. Following that, regeneration of new cells occurs. Sometimes, there is a malfunction in this regulated process which allows abnormal cells to continue to grow [2]. These abnormal cells may form localized primary tumors [1].

Tumors may form cancerous or non-cancerous tissue [3]. Cancerous tissue is known as malignant whereas non-cancerous is known as benign [3]. Malignant tumors have the ability to migrate to other nearby tissues and can travel to distant sites in the body to form new tumors; this process is called metastasis. There are various malignancies which form solid tumors. However, cancers of the blood, such as leukemias, generally do not. Nevertheless, when cancerous tumors are excised, they usually do not regrow, although in some situations they do. On the other hand, benign tumors remain localized and can sometimes have a large mass and become life-threatening [4]. For example, they can sometimes cause crowding of the normal structures inside the skull, leading to benign tumors in the brain [4,5]. Additionally, non-cancerous tumors can pose a threat to one’s health by pressing on vital organs [5]. Fayed has also noted that these non-cancerous tumors do not invade the neighboring tissues. In contrast, malignant tumors do invade neighboring tissues and are considered to be dangerous to one’s health [5].

The most common malignant tumor in women is breast cancer, which is the highest cause of female mortality globally [4]. Breast cancer development and progression are linked to several complicated anatomical changes and molecular processes. The present level of knowledge regarding the normal macro- and micro-anatomy of the human mammary gland has been addressed in the literature recently [6]. Moreover, it has been stated that the human mammary gland undergoes distinct development and differentiation from embryogenesis through to postmenopausal age [6]. However, even today, Sir Astley Paston Cooper’s (1768–1841) descriptions of mammalian breast dissections in 1840 are still cited to describe the anatomy of the breast [6]. Abrahams held that doctors need to have a good understanding of the anatomy of the breast in order to perform clinical breast exams that are effective [6]. The stages of breast development include pre-puberty, puberty, pregnancy, lactation-associated remodeling, and post-lactational and post-menopausal involution. Breast tissue differentiates and becomes more specialized as the mammary gland develops into an efficient milk-secretory organ during the pregnancy and lactation cycle (PLC). However, as multiparous females are susceptible to the PLC cycle, childbearing and nursing may offer some long-term protection against the onset of breast cancer [6].

Breast glandular tissue has a ductal epithelium that makes up 85% of cases and a lobular epithelium that makes up 15% of cases of breast cancer [7]. The malignant tissue matures within the duct or lobule (“in situ”) where it is asymptomatic and has a decreased likelihood of proliferating (metastasis). These in situ (stage 0) tumors may develop over time and spread to the neighboring lymph nodes (regional metastasis), other body organs (distant metastasis), or the breast tissue itself (invasive breast cancer) [7]. Breast cancer can grow rapidly in the female population, thus resulting in increased mortality rates. According to estimates, metastasis has caused 90% of cancer-related deaths [8]. In most cases, treatment is effective, especially when the breast cancer is discovered early. Radiation therapy, hormone therapy, and surgical removal of a tumor are frequently used in conjunction to treat breast cancer [9]. Other methods employed include chemotherapy and/or targeted biological treatment [9]. Such therapy can stop the growth and spread of cancer, thereby saving lives [9].

An increase in cancer survival rates has been achieved as a result of the discovery of anti-cancer drugs, such as anthracyclines (ATCs) [10,11]. Anthracyclines are frequently referred to as antibiotics and are generated from the Streptomyces bacteria [11]. Doxorubicin and daunorubicin are the two most important ATCs that are used therapeutically. As a kind of chemotherapy, they are used to treat a number of cancers, including breast cancer [11]. These substances operate by impeding DNA metabolism-related biological processes, which kills rapidly dividing cells [12]. The majority of anthracyclines are injected straight into the bloodstream, and common adverse effects include hair loss, nausea, vomiting, and a reduction in immune cell production. The most serious potential adverse side effect is dose-related cardiac tissue damage; however, current treatment protocols including Dexrazoxane has been created to minimize this risk [11,13].

Since the introduction of ATCs over 50 years ago, the survival rate of cancer in both genders has increased. Doxorubicin (Dox) is one of the ATCs that is used to treat various malignancies, including breast cancer [14,15]. However, the use of the chemotherapeutic agent Dox has resulted in a number of contraindications after its administration. The side effects of Dox may present in a matter of days (acute toxicity) or even years (chronic toxicity) following chemotherapeutic treatment. Dox-associated side effects include the development of heart disease as well as resistance to the treatment [16]. Therefore, the steps that are currently being taken to overcome these side effects include the investigation of alternative drugs, such as herbal drugs, as potential cancer therapeutics.

Several researchers have become interested in the use of herbal medicines because they are affordable, easily accessible, natural, and have a number of significant biological qualities [13,17,18]. In the United States (US), between 50 and 60 percent of cancer patients use nutrition or treatments made from various plant and animal components, either exclusively or concurrently with a traditional treatment strategy like chemotherapy and/or radiation therapy [19,20,21]. These include, to name a few, curcumin from turmeric, genistein from soybeans, tea polyphenols from green tea, resveratrol from grapes, sulforaphane from broccoli, isothiocyanates from cruciferous vegetables, and silymarin from milk thistle [22].

Of interest in this study is *Moringa oleifera* (MO), a medicinal tree with the potential to be an anti-cancer agent, as it is an antioxidant as well as an inducer for cell death in different cancer cell lines, such as HepG_2_ liver cancer cells [23]. The MO tree is small in size: approximately 5 to 10 m in height. It is cultivated all over the world due to its multiple utilities [24]. Every part of MO is used for nutritional and/or medicinal purposes. Besides being a good source of protein, vitamins, oils, fatty acids, micro-macro minerals, elements, and various phenolics, it is said to have anti-cancer, anti-inflammatory properties. The tree, MO, has also been shown to inhibit oxidation, act as an antibacterial agent, be hepatoprotective, and contain ulcer-healing properties, as well as diuretic, antiurolithiatic, and antihelmintic properties [24]. MO’s multiple pharmaceutical effects are capitalized on as a therapeutic remedy for various diseases in the traditional medicinal system [24].Various studies have investigated MO extracts in different cell lines. However, there is still insufficient information on the usage of the aqueous leaf extract of MO in breast cancer, especially with the use of the MCF-7 cell line as an in vitro model. Therefore, this review aimed to highlight the potential of MO aqueous leaf extract as an antiproliferative agent, mainly focusing on its potential to increase oxidative stress leading to apoptosis in breast cancer cells.

## 2. Cancer

**Figure 1:**
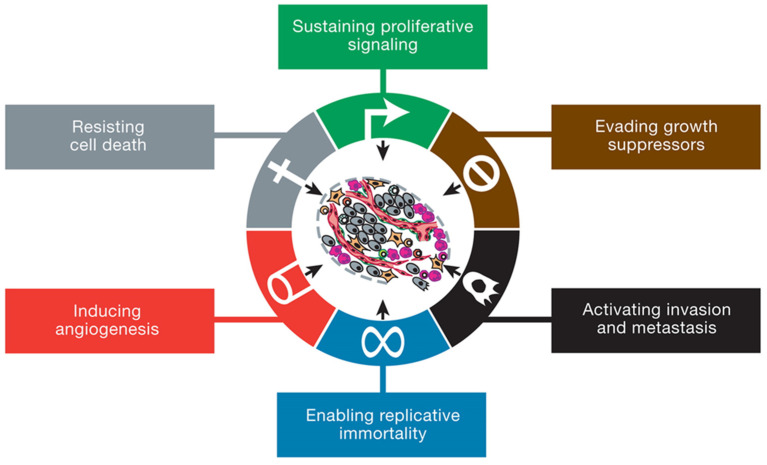
Hallmarks of cancer: These hallmarks help us to have a clear understanding of various human cancers. They include sustaining the signaling of cell proliferation, eluding growth suppressors, and cell death resistance. Additionally, induction of angiogenesis, avoiding destruction by immune cells, deregulation of cellular metabolism, genome instability, enabling of replicative immortality as well as tumor-promoting inflammation and the activation of invasion and metastasis [28].

Non-communicable diseases such as cancer contribute to high global morbidity and mortality rates [25,26]. Cancer is described as a disease state in which there is anomalous cell development and uncontrollable cell division [27]. There are several hallmarks of cancer that describe the abilities of cancer cells to permit its progression, including the ability to sustain signaling of cell proliferation, eluding growth suppressors, and cell death resistance (Figure 1) [28]. Additionally, the induction of angiogenesis during the cancer disease state and the ability of cancerous cells to avoid destruction by immune cells are also included as the hallmarks of cancer. Moreover, the deregulation of cellular metabolism in cancerous cells and the activation of invasion and metastasis during disease progression also form part of the characteristics of cancer [28,29,30]. The hallmarks of cancer continue to be the cardinal points when it comes to understanding the microenvironment of various cancers and providing a systematic basis to target different cancers for improved therapies [31,32,33].

## 3. Breast Cancer Epidemiology

**Figure 2:**
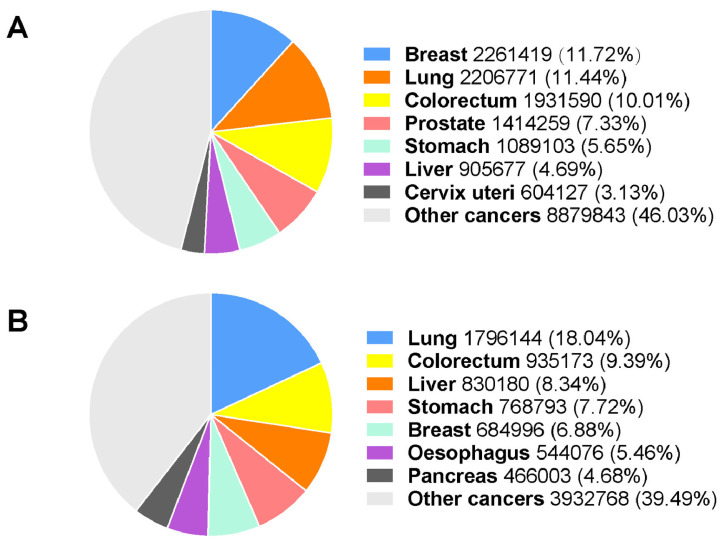
Estimates of the various cancer incidents and death rates worldwide, including breast cancer: (**A**) New cancer incidents estimated globally in 2020 with breast cancer included. (**B**) Global estimates of cancer mortality rates [37].

Cancer is the second leading cause of death worldwide, with breast cancer being the most common type of cancer affecting women [26]. An estimated 10 million cancer deaths were recorded globally in 2020 [34]. Of the estimated 19.3 million new cancer cases reported globally, breast cancer diagnosis in women accounted for 11.7% (Figure 2) [34]. It is predicted that more than 1.3 million untreated cases will succumb to the disease, unless the necessary course of action is taken [34]. South Africa (SA) has a total population of 59 million, with a reported 108,168 patients diagnosed with cancer in 2020 and 56,802 cancer-related deaths [35]. Almost a third of the recorded cancer cases (27.1%) were breast cancer [35]. Although the incidence of breast cancer is widespread across the world, its mortality rate, survival rate, and prevalence varies in various parts of the world because of different associated risk factors [36].

## 4. Breast Cancer Aetiology

Several studies have suggested that the onset of breast cancer is attributed to ovarian hormones such as oestrogens and progestogens [38,39,40]. Moreover, obesity before the onset of menopause decreases the risk of breast cancer and obesity post menopause increases the risk of breast cancer [39,41,42,43]. This is caused by the adipose tissue that acts as an oestrogen biosynthesis reservoir following menopause [43]. The high serum oestrogen levels and the intensified peripheral oestrogen site of production have been considered reasons for breast cancer development in overweight women following menopause [43]. Additionally, it has been shown that females with blood group A who are Rhesus positive experience a high risk of breast cancer development, whilst those who are Rhesus negative with blood group AB had the lowest breast cancer risk [36].

It has also been shown that inherited gene mutations of BRCA1, BRCA2, and PALB-2 elevate the risk of cancer greatly [1]. Females found to display mutations in these major genes are advised to consider surgical removal of the breasts; however, this should not be rushed because thorough decisions and procedure should be followed [1]. The early onset of breast cancer is common in females that harbor the mutations of BRCA1 with approximately 80% of them presenting with aggressive triple-negative tumors [44]. Additionally, 50% of adolescents and young adults who are diagnosed with cancer of the breast before the age of 30 present with mutations of BRCA1, BRCA2, and TP53 [44]. Moreover, the risk of developing breast cancer in adolescents and young adults is increased by the germline PALB-2 by at least 8-fold [44].

Exogenous hormones may also contribute to elevating the risk of breast cancer. These hormones may be found in oral contraceptives or through oestrogen replacement therapy [45]. The risk of breast cancer elevates in adolescents and young adults who use the above hormones, particularly in teenagers who carry germline BRCA1 mutations [44]. However, a study by Balekouzou and colleagues illustrated that the ovulatory menstrual cycle may protect against developing breast cancer and that breastfeeding decreases the risk of developing breast cancer in young adults as well as adolescents [44,46]. Other risk factors include the ingestion of a high-fat diet during childhood and adolescence and the consumption of alcohol, which contributes to the development of breast cancer by elevating oestrogen production [43,47,48,49,50,51]. It has also been outlined that low body mass index, as well as substantial weight gain in adolescents and young adults, elevates the risk of oestrogen receptor-negative breast cancer, and in contrast, the risk is decreased by vigorous exercise [44]. Furthermore, studies have indicated that currently employed breast cancer treatments lead to various side effects, e.g., cardiomyopathy and developing chemo-resistance [52,53,54]. In order to prevent the side effects of cancer treatments, the pathophysiology of breast cancer and the anti-cancer drugs’ mechanisms must be fully understood.

## 5. Breast Cancer Pathophysiology

Breast cancer development is a result of damage to the deoxyribonucleic acid (DNA) and mutations to the genes that can be influenced by exposure to oestrogen receptors, progesterone receptors, and human epidermal growth factor receptor 2 [55,56]. Cells with abnormal DNA are attacked by the immune system in cancer-free individuals. Failure to fight the rapid abnormal DNA growth leads to tumor growth. Reoccurrence of breast cancer is predicted on the basis of tumor markers [57]. Examples may include metastatic breast cancer occurring within a period of three years in patients who do not show any tumor markers or occurring ten years later following the first diagnosis and treatment in estrogen-receptor positive tumor patients [57]. Breast cancer can occur in two tissue types: ductal and lobular epithelium [58]. While most cancerous abnormalities of the breast originate from within the ductal epithelium, the cancerous cells may also arise within the lobular glands [58]. In other cases, DNA defects or pre-cancerous genes such as BRCA1 and BRCA2 may be inherited [55]. Therefore, a family history of breast cancer is considered to elevate the risk of development of breast cancer [55]. It is essential for women to screen for breast cancer constantly, especially if they come from a family with a history of breast cancer [59].

## 6. Breast Cancer Diagnosis

Breast cancer is diagnosed by screening a patient or conducting a diagnostic exam following an experience of a symptom [60]. Mammography has been proven to improve breast cancer detection as well as reduce mortality by 19%. It is recommended when a woman reaches the age of 45 [60]. Women who carry the mutations of BRCA1 and BRCA2 have a higher risk of developing breast cancer [61]. Therefore, an annual mammogram is required for individuals with a family history of cancer at the earliest age of 25 but not later than 40 years. Additionally, the utilization of magnetic resonance imaging (MRI) as an adjunct to mammography is suggested by the American Cancer Society for classifying and measuring the extent of breast cancer as well as the size of the tumor [60].

## 7. Breast Cancer Classification

The TNM classification system is used to categorize breast cancer according to various criteria [62]. The “T” in the classification system indicates the size of the tumor and the localization of the tumor. The total number of lymph nodes that have cancer are indicated by the “N” in the classification system. The “M” represents distant metastasis, and this shows if cancer has spread to other parts of the body. The reason for classifying is to choose the best treatment plan and specific treatments are effective depending on the type of breast cancer. The TNM factors were grouped by the American Joint Committee on Cancer into overall stages of cancer [62].

## 8. Breast Cancer Stages and Grades

The first stage (I) indicates that the cancer is still localized and has not spread [30]. Stage two (II) refers to the size of cancer that has increased but has not spread. The third (III) stage indicates that the cancer cells may have spread to tissues or lymph nodes. In the fourth (IV) (final) stage, cancer has spread to other vital organs within the body [3]. The subtype and grade of breast cancer distribution vary with age and the phenotypes are more aggressive in adolescents and young adults as compared to women that are older [44]. The Basal-like and HER-2 enriched tumors of the breast are the types of breast cancer commonly found in young women as compared to older ones [44]. In addition, pre-menopausal females presenting with invasive breast cancer have an increased risk of developing oestrogen receptor-negative, progesterone receptor-negative, and pathologic third-grade tumors [44]. Metastasis is a process whereby a primary tumor transforms into a distal secondary tumor [63]. In most malignancies, including breast cancer, epithelial-mesenchymal transition (EMT) is necessary for metastasis [63]. During EMT, cancer cells advance and develop the capacity to migrate as well as become active. The bonds in epidermal cells are broken down by the EMT program, allowing migration of cancer cells into new tissues [63]. However, in order for one to know the stage of cancer, the grade of cancer has to be specified [64]. This is a process of measuring how fast the growth of the cancer is and how the cells look when compared to the normal cells [64]. This will provide better knowledge of the type of treatment that can be administered to the cancer patient.

## 9. Current Cancer Treatment Therapies

Cancer is a disease state in which cells grow uncontrollably [65]. As discussed previously, the various clinical cancer stages are characterized by the type, size, and degree of metastasis, which forms the determining factor for the specific anti-cancer treatment strategy [66]. Various conventional treatment therapies can be used for breast cancer: chemotherapy, hormonal therapy, radiation, and surgery [7,67,68]. In addition, immunotherapy can also be included in treatment plan. Immunotherapy is when a patient utilizes their own immune system to fight cancer [69]. This treatment therapy boosts and changes how the patient’s immune system works to detect, locate, and fight the cancer cells [69,70]. Moreover, targeted therapy can also be used as a treatment for cancer. Targeted therapy can be utilized as monotherapy or combined with other treatments including radiation, chemotherapy, or surgery and can also be used as an adjuvant [70].

The most common surgical procedure employed for breast cancer is a mastectomy in which there is the removal of the entire breast. This includes all the breast tissue and, at other times, the surrounding tissues as well [71]. The breast-conserving surgery (Lumpectomy) can also be performed where only part of the breast tissue that contains the cancer is removed [71]. A patient who had a mastectomy does not need to undergo radiation therapy as compared to one who has performed a lumpectomy [71]. Radiation therapy is the treatment that destroys cancer cells with high-energy particles that include x-rays, electron beams, and protons.

Additionally, hormonal therapy is also used as a treatment for breast cancer. Oestrogen and progesterone hormones have been shown to affect some types of breast cancers as they help the tumors to grow [71]. However, hormonal therapy prevents the hormones from attaching to the protein receptors found in the breast cancer cells [71]. Hormone therapy can be administered as an adjuvant following surgery to reduce the reoccurrence of breast cancer.

Chemotherapy can also be utilized to treat breast cancer. These anti-cancer drugs may be injected into a patient’s vein or administered orally [72]. Chemotherapy may be recommended following surgery (adjuvant chemotherapy) to kill the remaining microscopic cancer cells or prior to surgery (neoadjuvant chemotherapy) to shrink a tumor’s size so it can be removed with a less extensive surgery [72]. If, following the neoadjuvant chemotherapy, cancer cells are still present, then more chemotherapy will be recommended (adjuvant chemotherapy) to reduce the recurrence of cancer [72].

**Figure 3:**
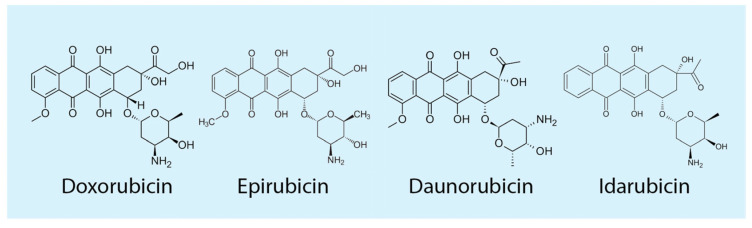
Anthracyclines (ATCs): There are various ATCs that are used as chemotherapeutic agents to treat different malignancies. They include Doxorubicin, Epirubicin, Daunorubicin, and Idarubicin [74].

Over the past decade, a significant improvement in cancer patient outcomes has been indicated due to the advancement of anti-tumor antibiotics such as anthracyclines (ATCs), Figure 3 [14]. These anti-tumor antibiotics change the DNA found in the cancer cells to prevent them from growing and multiplying [69]. According to Abdullah and colleagues, ATCs have become the cornerstone of chemotherapy for various cancers, especially since many ATCs are already effective and approved as anti-cancer drugs [15,73]. These include Daunorubicin, Epirubicin, Idarubicin, and Doxorubicin [15,73,74].

Of interest in this study is Doxorubicin (Dox), initially isolated in the 1950s from the soil bacterium Streptomyces peucetius [75]. Dox is also referred to as Adriamycin (Figure 3). The United States Food and Drug Administration (FDA) approved the chemotherapeutic drug Dox, which is frequently used to treat various cancers such as lung cancer, stomach, Hodgkin’s lymphoma, ovarian cancer, and breast cancer [11].

**Figure 4:**
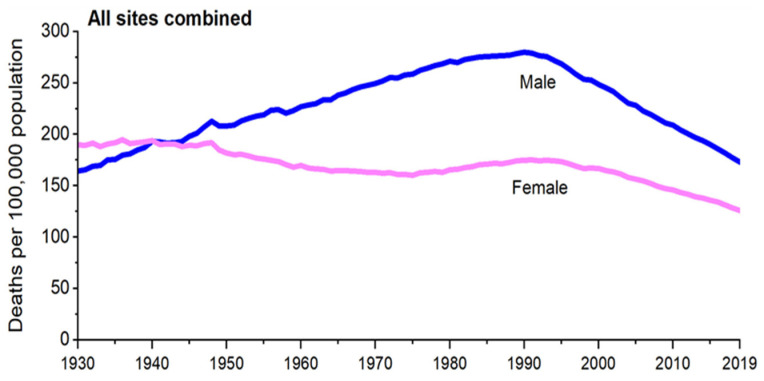
Improvement of cancer patient outcomes: the number of cancer death cases has decreased globally over the years as a result of the administration of chemotherapeutic agents (ATCs) [77].

Dox has improved the cancer survival rate from 40% in 1971 to more than 70% in 2016 for both males and females (Figure 4) [10,11,76]. However, it has been shown that administering Dox in high doses can permanently damage the heart of patients [72]. As a result, cumulative doses are recommended when this drug is administered [72].

**Figure 5:**
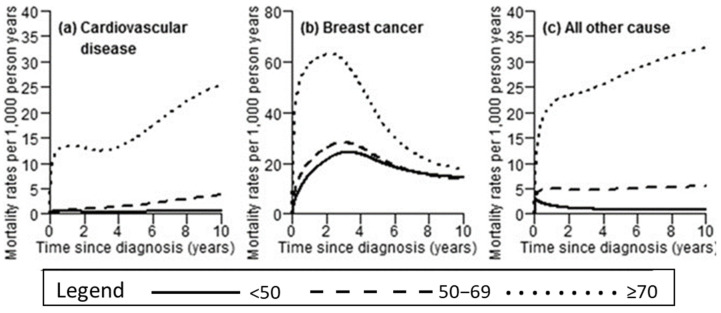
Administration of ATCs improves cancer patient outcomes at the expense of increasing cardiovascular deaths [78,79].

Unfortunately, the use of Dox is associated with dose-dependent side effects (Figure 5) [13]. This includes the development of oedema which leads to heart failure, adverse effects on the liver, nerve damage, and an elevated risk of developing leukemia as well as chemo-resistance [11]. Breast cancer remains incurable, with several side effects associated with chemotherapeutic regimens. However, the pathophysiology and the mechanism of action of breast cancer drugs require better understanding in order to circumvent common side effects and to reduce cancer cell proliferation.

## 10. Mechanism of Action of Dox

Doxorubicin’s mechanism of action involves its ability to interject within the base pairs of DNA [12]. This causes the DNA strands to break and inhibits the synthesis of both DNA and RNA [12]. Dox causes the inhibition of the enzyme Topoisomerase II, the normal function of which is to untangle the supercoils and make space for new strands of DNA to be created [80]. However, in a cancer disease state, the Dox inhibition therefore causes damage to DNA (disrupting adenosine triphosphate (ATP) production), which leads to the induction of cell death [12,13,81,82]. Furthermore, the combination of Dox with iron limits DNA synthesis by causing free radical-mediated oxidative damage [12].

## 11. Role of Oxidative Stress in Cancer

**Figure 6:**
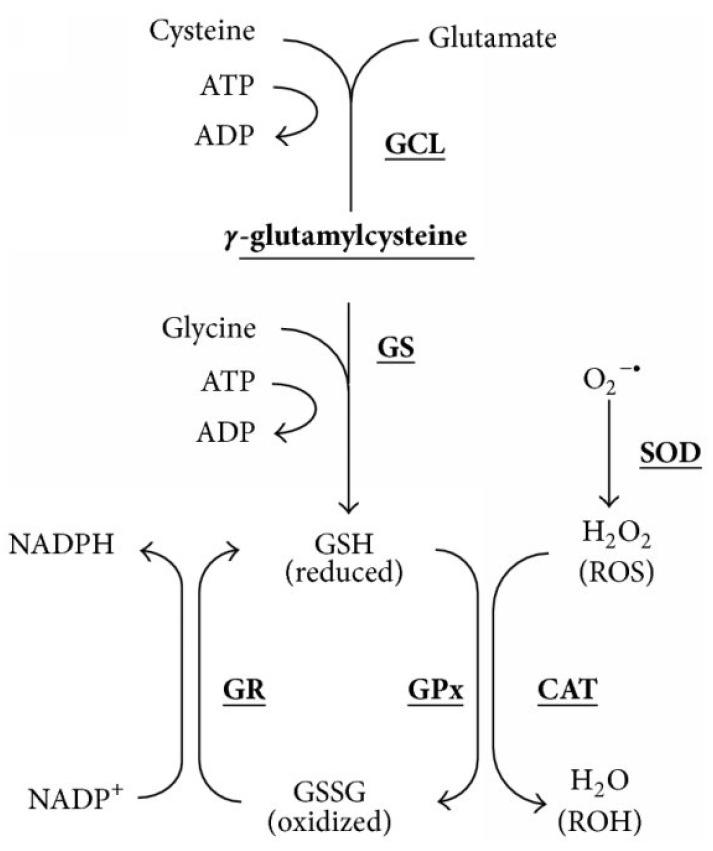
Glutathione reduction-oxidation reaction: Glutathione (GSH) consists of three amino acids. Glutathione peroxidase (GPx) is involved in the detoxification of ROS. The production of hydrogen peroxide (H_2_O_2_) results from the conversion of superoxide anion (O_2_^−^) by superoxide dismutase (SOD). An oxidized form, glutathione disulfide (GSSG), is produced and recycled back to GSH utilizing a glutathione reductase enzymatic reaction. A cofactor, Nicotinamide adenine dinucleotide phosphate (NADPH), is required for a redox reaction to occur [92].

Oxidative stress is a cellular process that underpins a variance in the production of reactive oxygen species (ROS) and endogenous antioxidants, potentially leading to cellular damage [83,84]. Cellular ROS are produced endogenously during mitochondrial oxidative phosphorylation and may also be generated during cellular interactions with xenobiotic compounds [85]. Endogenous antioxidants are required to mitigate ROS-mediated injury [86]. Antioxidants such as glutathione (GSH) are compounds that scavenge free radicals (superoxide, hydrogen peroxide, and hydroxyl anion) by maintaining oxidation-reduction (redox) equilibrium (Figure 6) [86,87,88,89]. GSH comprises amino acid cysteine, glutamic acid, and glycine (Figure 6), which modulates the cellular redox status [88,90,91].

**Figure 7:**
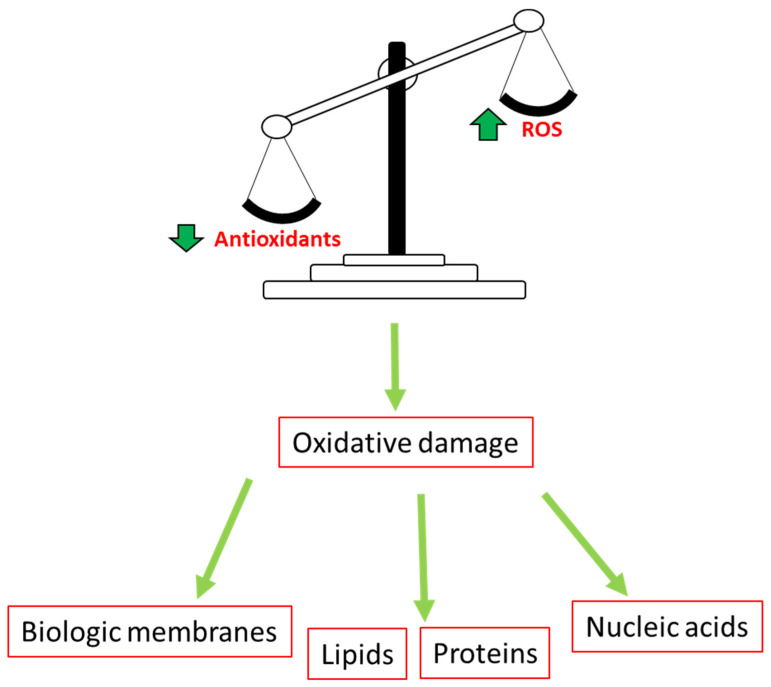
Mechanism of oxidative stress: Increased levels of ROS trigger activation of stress-related pathways. The imbalance between free radicals and antioxidant levels in the body leads to oxidative damage to cells [98]. The oxidative damage causes protein lesions. This damage may lead to the induction of apoptosis, nuclear and mitochondrial DNA damage, or lipid peroxidation. Adapted from [99].

The decreased level of GSH, accompanied by an increase in ROS, may lead to the induction of oxidative stress, including the direct oxidative impairment of DNA, lipids, and proteins (Figure 7) [93,94]. Damage to DNA has been hypothesized by Lee and colleagues to take part in the initiation of carcinogenesis of the breast [94]. In contrast, an increase in GSH levels may protect breast cancer cells against developing chemotherapeutic resistance [95]. Nuclear factor erythroid 2-related factor 2 (Nrf2) is a transcription factor that regulates cellular antioxidant expression, including GSH [96]. Harvey and colleagues demonstrated that Nrf2 maintains the homeostasis of GSH by altering the de novo synthesis and modulating the redox state of GSH [91]. In addition, Nrf2 protects the cells from oxidative stress [91]. The imbalance between ROS and antioxidants leads to direct oxidative impairment (Figure 7) [94]. Lee and colleagues also suggested that ROS production and the resultant oxidative stress are the major aetiology leading to various cancers, including breast cancer development.

Elevated oxidative stress may also result in cytotoxicity, cause the inhibition of cell proliferation, and cause apoptosis or necrosis. In contrast, a decline in oxidative stress may lead to DNA damage, mutation, and proliferation of cells and may eventually cause carcinogenesis [97].

## 12. Antioxidants and Breast Cancer

Cells fail to maintain their own homeostasis because of the variance in oxidative stress [100]. This leads to the induction of DNA damage and therefore cell death. Irreversible damage to the cells leads to the development of cancer. Oxidative stress induces pro-apoptotic pathways leading to cell death [100]. Increased ROS levels in cancer cells as compared to normal cells are considered to be the cause of carcinogenesis as it sustains malignant phenotypes and promotes proliferation of cells, as well as metastasis and angiogenesis [101]. The cancer cells thrive on low levels of oxygen, and evidence has shown that oxidative stress and lipid peroxidation are associated with the development of breast cancer [102,103]. Natural products such as flavonoids and phenolics have been shown to be effective in cancer as they suppress carcinogenesis (both early and late stage) [104]. Studies have shown that antioxidants may cause apoptosis in tumor cells but also inhibit the proliferation of cancer cells and spare normal cells [92,105,106,107]. Tumor cell hyperproliferation is a result of the increased production of ROS [101]. The cancer cells accomplish this by raising their oxidant status for ROS-driven proliferation optimization, concurrently avoiding the threshold of ROS that would lead to apoptosis and ferroptosis [101]. Reports have been made that mitochondrial ROS leads to the promotion of cell death. Meanwhile, NOX-generated ROS promotes the proliferation and migration of cells [103]. Xue and colleagues demonstrated that ROS regulates chemoresistance and chemosensitivity of cancer to various tumor drugs [108]. Additionally, Nrf2 plays the same role as ROS of resulting in chemoresistance of cancer drugs [108].

## 13. Nrf2 and Cancer

The transcription factor Nrf2 is considered to be a cellular protector (for both normal and cancerous cells) that stimulates how genes express themselves [109]. It performs the double role of leading to the inhibition of cancer development and also promoting the progression of cancer, as well as resistance to chemotherapy [108]. It has sequences of antioxidant response elements in the promoter region [109]. The hyperactivation of Nrf2 has been shown to induce proliferation as well as overgrowth of tumor cells. This prevents tumorous cells from going through apoptosis and therefore causing the cells to be resistant to both chemotherapy and radiotherapy [109]. The Nrf2 gene has been demonstrated to be stabilized by K-RAS and c-MYC oncogenes which induce the production of intracellular ROS [103]. Moreover, it has been shown that mutant p53, which contributes to the proliferation of cancer and metastasis, causes the activation of Nrf2 gene transcription [110,111]. It has also been reported that targets of the gene Nrf2, including heme oxygenase 1 (HMOX1), initiate the development of cancer as they prevent the oxidative stress effect in cells that are transformed [103,112,113]. However, the HMOX1 target gene is repressed by p53 [110].

## 14. Role of Tumor Protein p53 in Cancer

Apoptosis is known as a vital defense mechanism for different processes such as normal cell turnover and how the immune system functions [114]. It can be triggered by different stimuli and conditions and not all cells can succumb to the same stimuli [114]. Chemotherapeutic agents induce apoptosis through the p53 dependent pathway [114]. Therefore, apoptosis is dependent on the therapeutic efficiency of anti-cancer drugs in targeting cancer cells. Since the nuclear transcription factor, p53, regulates the fate of the cell in response to damage to the DNA, it is necessary to have a therapeutic approach that activates the p53-mediated pro-apoptotic pathway. It has a principal role in controlling antioxidant gene expression [103]. Moderate levels of ROS lead to the inhibition of p53, whilst high levels encourage the promotion of its expression [103]. The p53 tumor suppressor (TP53) also regulates important processes such as cell metabolism, progression of cancer cells, and senescence in response to different stresses on the cell [115]. The p53 gene has been shown to mutate in various tumors, including breast cancer [115,116]. In some cases, these p53 mutations may show phenotypes that are chemo-resistant [117]. In cancer cells, there is downregulation of pro-apoptotic proteins, such as Bax, and the overexpression of the anti-apoptotic protein, Bcl-2. This may result in cancer cell survival. Moreover, the TP53 function may be dysregulated by a number of cancer cells that evade apoptosis. The induction of cell cycle arrest or apoptosis depends on how extensive the DNA damage is [117]. The cell cycle arrest that is mediated by the p53 gene permit the cells that have their DNA damaged to be repaired. However, when there is DNA damage to the cells, p53 exercises its pro-apoptotic function to remove cells with extreme DNA damage and prevent the transferal of impaired DNA to daughter cells. Therefore, p53 is well known for its ability to maintain the integrity of genomes [117].

## 15. Apoptosis (Programmed Cell Death) on Cancer Development

**Figure 8:**
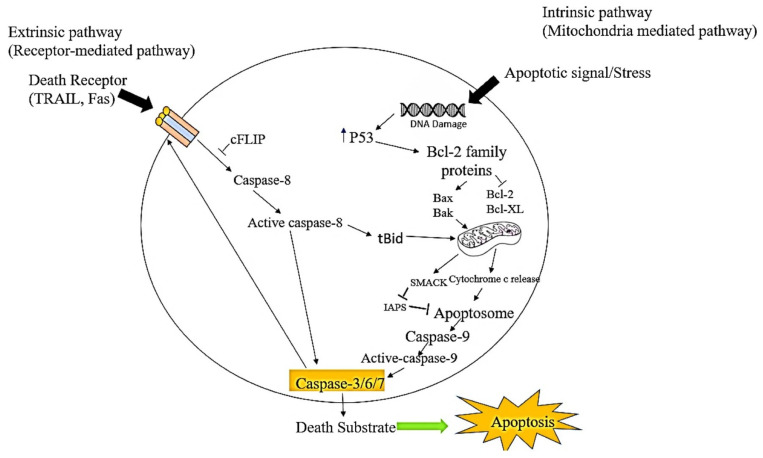
The intrinsic and extrinsic mechanism of apoptosis: Increased oxidative stress in cells or the activation of extracellular ligands induces DNA damage and mitochondrial impairment. The release of cytochrome c from the mitochondria forming an apoptosome with APAF-1, procaspase-9, and ATP initiates the caspase cascade [120,130].

Apoptosis, referred to as programmed cell death, is a self-destructive process of cells [118]. The apoptotic pathway is activated through increased ROS production and DNA damage [119]. Two signaling pathways activate cellular death, namely the intracellular (mitochondrial) and extracellular (death receptor) apoptotic pathways [13,119]. The intracellular signals are characterized by DNA damage, whilst the extracellular signals have cytotoxic T cells that produce death-inducing signals [120]. In cancer, there is an inhibition of apoptotic pathways (Figure 8) through the up-regulation of anti-apoptotic proteins (Bcl-2) and the downregulation of the pro-apoptotic proteins (Bax), resulting in intrinsic resistance to chemotherapy [119]. Mitochondrial depolarization is one of the principal intrinsic mechanisms by which apoptosis is induced. In a study performed by Shabalala and colleagues, Dox activated p53, a tumor suppressor protein. The activation of p53 increased the expression of the Bcl-2-like protein 4/B-cell lymphoma 2 (Bax/Bcl-2) ratio. This led to increased mitochondrial membrane permeability, releasing cytochrome c and aiding the activation of apoptosis through elevated caspase-3 expression (Figure 8) [121]. Dox has also been shown to inhibit topoisomerase II, an enzyme required by cancerous cells that promotes division and proliferation by intercalating with cellular DNA [122]. Additionally, Dox has also been demonstrated to induce apoptosis by activating the enzyme Poly (ADP-ribose) polymerase-1 (PARP-1) [123].

The PARP-1 protein is involved in the regulation of developmental processes such as the signaling of DNA damage [124,125]. It is also responsible for the proliferation, as well as differentiation, of cells. The PARP-1 protein detects and repairs DNA damage [126,127]. Moreover, it has been shown that the activation of the PARP-1 protein may play a role in inducing side effects like Dox-induced cardiotoxicity [128]. However, there is a need for alternative treatments such as herbal compounds that have the potential to activate apoptosis and elicit minimal side effects in various cancers [129].

## 16. The Potential of Herbal Drugs in Chemotherapeutic Regimens

Over the years, the interest in exploring alternative treatment modalities has escalated [131]. People have utilized herbal medicines to overcome and prevent certain diseases, particularly in patients with medical conditions such as type 2 diabetes mellitus, breast cancer, and human immunodeficiency virus [17,132,133]. Many traditional medicines have been investigated. Their antiproliferative effects were seen in different cancer cells, e.g., Pinocembrin, a herbal drug that has demonstrated anti-cancer properties by increasing apoptosis and necrosis in breast cancer cells [13]. *Sutherlandia frutescens*, also known as cancer bush, is often utilized by traditional healers in SA to treat various sicknesses, including breast cancer [134,135].

Plant-based therapy, such as the use of flavonoids, has been extensively investigated for its protective properties against the toxicities inferred by chemotherapy [73]. Flavonoids are known polyphenolic compounds with antioxidant, anti-inflammatory, and anti-carcinogenic as well as cardio-protective properties [73]. The use of plants for their therapeutic potential against various diseases has significantly increased over the years. Recently, natural products contributed to the production of medicinal agents that treat both chronic and acute human diseases [136]. Interestingly, studies have shown that plant-based medicines possess several biological activities, including anti-cancer properties [13,17,18]. However, the biological activities regarding the cancer treatment potential of several plants that are used traditionally are yet to be explored. Therefore, the use of a plant such as *Moringa oleifera* (MO) in traditional medicine in SA requires extensive investigation for its biological properties to be fully elucidated.

## 17. *Moringa oleifera*

**Figure 9:**
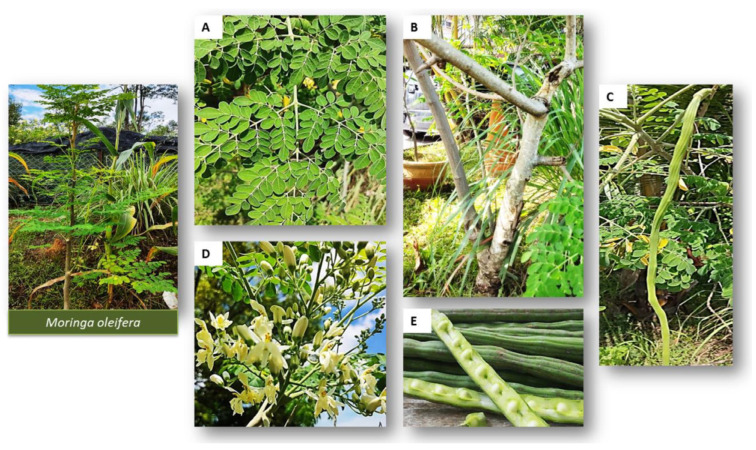
The various parts of *Moringa oleifera* tree (**A**) Leaves, (**B**) Stem and Bark, (**C**) Pods, (**D**) Flowers and sepals as well as (**E**) seeds [141].

*Moringa oleifera* (MO), from the family Moringaceae, is a tree native to India (Figure 9) and is found throughout SA [137,138]. It is commonly known as horseradish or the drumstick tree [138,139,140]. MO is considered to be the “powerhouse” of essential nutrients. The leaves are rich in calcium, copper, magnesium, potassium, zinc, and iron. It contains vitamins A, B, C, D, and E [138].

**Figure 10:**
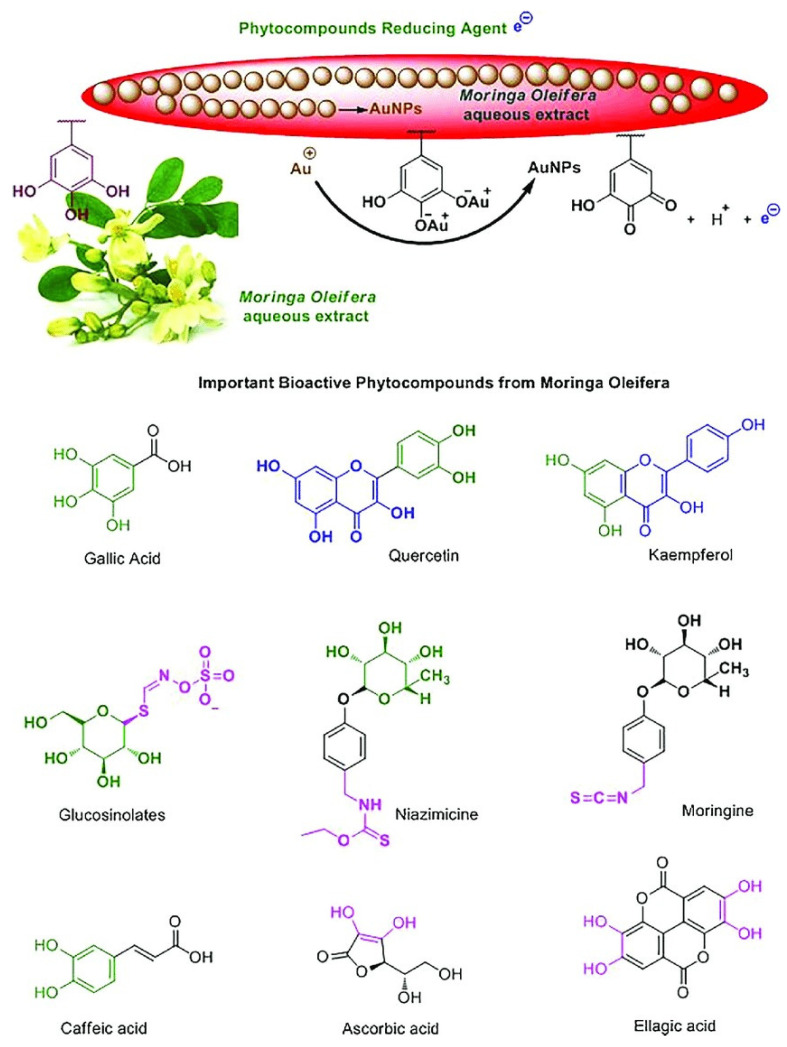
Chemical structures of bioactive compounds of MO. There are various compounds found in MO that make it a good chemotherapeutic agent. This includes Niazimicin as well as Kaemferol and Quercetin [146,151]. Moreover, the various colors demonstrate compounds that contained the water soluble hydroxyfunctional group which were responsible for the reduction of gold ions and the stabilization of gold nanoparticles (AuNPs).

MO has been traditionally used to treat viral and bacterial infections, inflammation, and hyperglycemia [142]. It has been used traditionally in many countries; for example, Nigerians use MO to treat fertility disorders in females and increase the male fertility capacity [143]. The seeds of MO were also proven to purify water in African countries, such as Ethiopia [144]. MO contains niazimicin, a thiocarbamate that suppresses cancer cell proliferation (Figure 10) [145,146]. It also contains benzyl isothiocyanate, a compound that increases intracellular ROS and apoptosis, emphasizing the anti-cancer potential of MO [145,147,148,149,150].

MO has an abundance of phytochemicals present in the leaves, pods, and seeds, including sterols, flavonoids, and anti-cancer agents such as glucosinolates, isothiocyanates, and glycosides (Table 1) [138]. MO also contains Zeatin, which is an anti-aging compound. Zeatin has anti-cancer properties and is a good antioxidant [152]. It also has bioactive compounds, such as saponins and tannins, which have anti-cancer properties and alkaloids (Table 1), which have the potential of being cardiac stimulants [152,153]. This means it could possibly prevent the cardiac problems induced by Dox [152].

In addition, studies showed that the high antioxidants and bioactive compounds found in MO play an important role in preventing the heart damage inferred by chemotherapeutic agents such as Dox [155]. According to Gopalakrishnan et al. (2016), MO can also be used to treat malnutrition in children. It is described as the “miracle tree” because it is fast-growing, multi-purpose, and drought tolerant, as well as containing the above-mentioned nutritional and medicinal properties [138]. However, further research on MO and Dox herb–drug interaction are required as they influence cytochrome P450 enzymes, for example, CYP3A4, CYP1A2, and CYP2D6 in liver cells [156,157,158]. Asare and colleagues also indicated that supplementing the leaf extract of MO can become toxic if the levels are higher than 3000 mg/kg body weight but are considered safer if below 1000 mg/kg [159].

## 18. Anti-Cancer Properties of *Moringa oleifera*

Studies have demonstrated that MO can be used as an anti-cancer agent at established concentrations (Table 2). Both solvent and soluble leaf extracts of MO have been demonstrated to have effective anti-cancer properties [138]. Furthermore, studies have indicated that the antiproliferative effect of MO may be attributed to its ability to elevate oxidative stress, leading to DNA fragmentation and induction of apoptosis in A549 lung cancer cells [18,82,160]. Luo and colleagues found that phytochemicals in MO induced apoptosis by activating p53 in ovarian cancer cells [161]. In addition, MO induced apoptosis by causing DNA damage in hepatocellular carcinoma cells (HepG_2_) [162]. MO leaves are regarded as a good free radical scavenger because they contain quercetin-3-0 glucoside and kaempferol-3-0 glucoside (Figure 10) [143].

MO has also been demonstrated to increase the levels of antioxidants leading to decreased oxidative stress [169]. According to Suphachai (2014), both methanol and dichloromethane extracts of MO can suppress the growth of human colorectal adenocarcinoma cells (Caco-2) as well as breast adenocarcinoma (MCF-7) cells [23]. This was further supported by Karim and colleagues as well as Wang and colleagues, who found a reduced nuclear factor kappa B (NFKB) expression in MCF-7 breast cancer cells using the dichloromethane extract, which subsequently caused a decline in ROS formation [170,171,172]. Although the potential of MO has been scientifically proven to possess an anti-cancer, antioxidant, and anti-inflammatory effect on various other cancer cell types, future studies that include toxicity levels in in vivo studies and clinical trials should be considered. However, this current study investigates the anti-cancer potential of the aqueous leaf extract of MO, looking at its antioxidant and apoptotic effect on MCF-7 breast cancer cells. Moreover, additional studies are required to indicate if it is safe and effective in humans.

## 19. Conclusions and Future Perspectives

Based on the current literature, MO can be used for multiple purposes, such as purifying water, and consumed as a source of various nutrients. In addition, MO can treat various diseases and can also be used as an anti-cancer drug in various cancer cell lines. Most of the biological activities aided by MO are caused by the high flavonoid, glucosides, and the glucosinolates it contains. Additionally, previous research has shown MO’s ability to induce cell death and display its antioxidant potential in various malignant cell lines. Moreover, the different parts of MO, as well as various solvent extracts, have been investigated. However, there is a still lack of information on the antiproliferative mechanism of MO aqueous leaf extract in breast cancer. As there were investigations previously performed on the pathophysiology of breast cancer and the potential of MO as an antiproliferative agent, more research can be conducted to supplement the present literature. Further investigations into the specific bioactive compounds present in MO playing a role in inhibiting cancer proliferation can be conducted. Another investigative model, such as an in vivo model which is a multicellular system, can also be utilized.

## Figures and Tables

**Table 1:** The phytochemicals present in MO. The leaves of MO have more flavonoids and Saponins present as compared to other compound [154].

Constituents	Aqueous Extract
Alkaloids	++
Flavanoids	+++
Saponins	+++
Carbohydrates	++
Tannins	+
Steroids	++
Glycosides	+
Gums and mucilage	++
Lignin	ND
Phenols	+
Fixed oils and fats	++
Amino acids and proteins	++

Note: + = Low relative presence of compound ++ = Moderate abundance of compound +++ = Relative abundance of compound ND = Not detected.

**Table 2:** *Moringa oleifera’s* anti-cancer effects.

Experimental Model	MO Dose	Experimental Outcome	Proposed Mechanism	References
Human B-lymphocyte plasmacytoma (U266B1 cell line)	Methanol extract IC_50:_ 0.32 μg/mL	Increased cytotoxic activity	Inhibition of cell proliferation	Adapted from [163]
Lung cancer (A549 cell line)	Soluble cold distilled water extract 200 μg/mL	Demonstrated anti-cancer activity by reducing the expressions of AKT, NFKB, ERK, and cyclin D1	Induced apoptosis by activating caspases	Adapted from [18]
Lung cancer (A549 cell line)	Water-soluble extract IC_50_: 166.7 μg/mL	Reduced levels of GSH, induction of DNA damage as a result of decreased levels of PARP-1 and Nrf2	Apoptosis induced by activation of caspases	Adapted from [160]
Colorectal cancer (CRC) cell lines T84, HCT-15, SW480 and HT-29	Ethanolic seed extract IC_50_: 0.001 μg/mL	Caspases 9, 8, and 3 overexpression and elevated the production of ROS	Induced apoptosis by autophagy	Adapted from [164]
Pancreatic cancer (Panc-1 cell line)	Aqueous leaf extract 0.75 mg/mL	Reduced the p65 expression	Inhibition of cell proliferation	Adapted from [165]
Breast adenocarcinoma (MCF-7) and epithelial breast cancer cell line (MDA-MB-231)	Crude methanolic leaf extract 50 and 25 μg/mL	Decreased cell growth	Apoptosis induced in a time- and dose-dependent manner	Adapted from [166]
Cervical cancer (HeLa cell line)	Methanol leaf extracts IC_50_: 70 μg/mL	A decrease in cell viability with increased apoptosis	Apoptosis induced by DNA fragmentation	Adapted from [167]
Hepatocarcinoma (HepG_2_) and breast adenocarcinoma (MCF-7)	Dichloromethane extract 100 μg/mL	Demonstrated anti-cancer activity by reducing mitochondrial membrane potential, cell viability, and increasing DNA damage	Induced apoptosis by up-regulating Bax proteins	Adapted from [82]
Hep2 human epidermoid	Methanol extract 200 μg/mL	Induced DNA fragmentation	Induced apoptosis by elevating ROS	Adapted from [168]

## Data Availability

Data sharing is not applicable to this article as no new data were created or analyzed in this study.

## Abbreviations

ATC, Anthracyclines; ATP, Adenosine triphosphate; CVD, Cardiovascular Disease; CCM, Complete culture media; DNA, Deoxyribonucleic acid; Dox, Doxorubicin; Globocan, Global Cancer Observatory; GSH, Glutathione; MO, *Moringa oleifera*; Redox, Oxidation-reduction; ROS, Reactive oxygen species; WHO, World Health Organization; RNA, Ribonucleic acid; SA, South Africa; SD, Standard deviation; SOD, Superoxide dismutase; NADPH, Nicotinamide adenine dinucleotide phosphate; Nrf2, Nuclear factor erythroid 2-related factor 2; GSH, Glutathione.
